# Toward improved statistical methods for analyzing Cotinine-Biomarker health association data

**DOI:** 10.1186/1617-9625-9-11

**Published:** 2011-10-03

**Authors:** Tulay Koru-Sengul, John D Clark, Lora E Fleming, David J Lee

**Affiliations:** 1 Department of Epidemiology and Public Health at Leonard Miller School of Medicine, University of Miami, Miami, Florida, USA; 2 Sylvester Comprehensive Cancer Center at Leonard Miller School of Medicine, University of Miami, Miami, Florida, USA; 3 Department of Internal Medicine, Kaiser Permanente, Los Angeles, California, USA; 4 European Centre of Environment and Human Health (ECEHH), Peninsula College of Medicine and Dentistry, Truro, Cornwall, UK

## Abstract

**Background:**

Serum cotinine, a metabolite of nicotine, is frequently used in research as a biomarker of recent tobacco smoke exposure. Historically, secondhand smoke (SHS) research uses suboptimal statistical methods due to censored serum cotinine values, meaning a measurement below the limit of detection (LOD).

**Methods:**

We compared commonly used methods for analyzing censored serum cotinine data using parametric and non-parametric techniques employing data from the 1999-2004 National Health and Nutrition Examination Surveys (NHANES). To illustrate the differences in associations obtained by various analytic methods, we compared parameter estimates for the association between cotinine and the inflammatory marker homocysteine using complete case analysis, single and multiple imputation, "reverse" Kaplan-Meier, and logistic regression models.

**Results:**

Parameter estimates and statistical significance varied according to the statistical method used with censored serum cotinine values. Single imputation of censored values with either 0, LOD or LOD/√2 yielded similar estimates and significance; multiple imputation method yielded smaller estimates than the other methods and without statistical significance. Multiple regression modelling using the "reverse" Kaplan-Meier method yielded statistically significant estimates that were larger than those from parametric methods.

**Conclusions:**

Analyses of serum cotinine data with values below the LOD require special attention. "Reverse" Kaplan-Meier was the only method inherently able to deal with censored data with multiple LODs, and may be the most accurate since it avoids data manipulation needed for use with other commonly used statistical methods. Additional research is needed into the identification of optimal statistical methods for analysis of SHS biomarkers subject to a LOD.

## Background

A biomarker is a laboratory measure of a biological process [1]. The lowest quantity of a biomarker that can be distinguished from the lack of that biomarker is the biomarker's limit of detection (LOD), below which the level of biomarker cannot be accurately measured. One important yet unresolved issue in analyzing biomarker data arises when biomarker measurements fall below the LOD (i.e. "non-detects", "left-censored").

Statistical analyses of data that include biomarker measurements below the LOD are complicated since precise quantitative levels cannot always be determined [1]. In all analyses involving biomarkers with a LOD, researchers working with biomarker data inevitably have to deal with data containing non-detects, and must decide how to combine non-detects with values above the LOD for analysis. The choice of an appropriate strategy for dealing with data affected by LODs requires an understanding of both experimental and statistical procedures. Until now, the common practice has been to impute (i.e. substitute a single value, such as a half of the detection limit, for each measurement below the LOD), and to then conduct the analysis under the assumption that the imputed values are the actual observed values [2-4]. This assumption may be invalid, leading to biased results, especially when trying to predict small exposure-health outcome associations [5,6].

Analytic issues with secondhand smoke (SHS) exposure biomarkers (e.g. cotinine, 4-(methylnitrosamino)-1-(3-pyridyl)-1-butanol (NNAL) [NNAL]) arise due to a large percentage of measurements below the LOD. Serum cotinine, a metabolite of nicotine, is widely used in research as an objective measure of recent tobacco smoke exposure. The use of traditional statistical methods to analyze serum cotinine measurements often introduces bias into the study results due to measurements falling below the LOD, potentially affecting the accuracy of analytic results and validity of study conclusions [1-4]. Furthermore, this bias may be greatest in studies investigating the health effects of tobacco smoke exposures at very low exposure levels, a situation where the subjects' cotinine levels are more likely to be undetectable. Therefore, the impact of low-level SHS exposure on human disease may be underestimated using traditional statistical methods for handling biomarkers below the LOD [1,2].

The purpose of our research was to demonstrate and compare the performances of commonly used statistical methods as a case study for analyzing associations between serum cotinine measurements with non-detects and levels of the inflammatory marker homocysteine using data from the 1999-2004 National Health and Nutrition Examination Surveys (NHANES) [7].

## Methods

This project's data consisted of adults 18 and older participating in the 1999-2004 NHANES, a cross-sectional study designed to assess the health and nutritional status of adults and children annually in the United States (n = 31,126). The continuous NHANES survey combines interviews and physical examinations; the resulting data can be pooled across multiple years. Anonymous survey data and related documents were obtained from the NHANES website http://www.cdc.gov/nchs/nhanes.htm.

Subjects included in this analysis were non-smoking adults age 20 years and older as defined by a serum cotinine level less than or equal to 3.08 ng/mL or by self report of not smoking within the past 5 days. This particular cotinine threshold for adults has reportedly a sensitivity of 96.3% and a specificity of 97.4% for differentiating smokers from non-smokers [8]. The subjects included in this analysis had detectable or undetectable serum cotinine levels and complete information on additional variables of interest (i.e. homocysteine, age, gender, race/ethnicity, and SHS exposure) in order to focus on the issue of properly handling left-censored serum cotinine data (n = 9,488).

In the NHANES surveys, serum cotinine was assessed using an isotope dilution high performance liquid chromatography atmospheric pressure chemical ionization tandem mass spectrometric method [9]. The laboratory test detection limit for serum cotinine in NHANES was 0.05 ng/mL for 1999-2000 and part of 2001-2002. Improvements in the laboratory testing methods reduced the LOD to 0.015 ng/mL; this lower detection limit was used for the 2001-2002 and the 2003-2004 NHANES. For the 1999-2000 cycle, 37.7% of values were below the LOD (0.05 ng/mL), while 22.6% were below the LOD for the 2001-2002 cycle (either 0.015 ng/mL or 0.05 ng/mL) and 16.9% were below the LOD for the 2003-2004 cycle (0.015 ng/mL). Plasma homocysteine was analyzed using an automated fluorescence polarization immunoassay [9].

We compared commonly used statistical methods (Table 1) as a case study for analyzing serum cotinine with measurement values below the LOD using parametric and non-parametric techniques: complete case analysis, single and multiple imputation methods, "reverse" Kaplan-Meier method, and logistic regression models [2].

**Table 1:** Characteristics and Comparisons of the Statistical Methods Used for Analysis of Biomarker Data with Limits of Detection

Method	n used (% total)	n below LOD	Transforms censored data to categorical data	Replaces censored values with imputed value	Inherently able to deal with multiple LOD?
Complete Case	5,865 (62)	0	No*	No*	No*
Single Imputation	9,488 (100)	3,623	No	Yes	No
Multiple Imputation	9,488 (100)	3,623	No	Yes	Yes
Logistic regression	9,488 (100)	3,623	Yes	No	Yes
"Reverse" Kaplan-Meier	9,488 (100)	3,623	No	No	Yes

LOD: limit of detection

*Excludes censored data

### Methods of Handling Left-Censored Data

Complete-case analysis is a method of analysis that incorporates only subjects with serum cotinine values above the LOD. This is a widely used method due to its simplicity, but is highly inefficient since it reduces sample size and produces bias and loss of precision in the estimation [10].

Imputation is the practice of replacing undetectable serum cotinine with "plausible" values [11]. After imputation, data can be analyzed as if imputed values were actual observed values. In single imputation, each non-detect value is replaced with an estimate, but this does not account for the sampling variability produced by imputed values. Single imputation generally results in the underestimation of variance, which directly affects confidence intervals and statistical tests. In our analysis, we performed single imputation analyses substituting one of three different commonly used substitution values for non-detect cotinine measurements: 0, LOD, and LOD/√2. Depending on the LOD of the cotinine measurement method, single imputation values changed when the LOD changed across survey cycles (i.e. 0.05 ng/mL for 1999-2000 and part of 2001-2002; 0.015 ng/mL for part of 2001-2002 and 2003-2004).

Multiple imputation is a simulation-based approach which can provide a good solution to missing data problems; and it has been used extensively with complex national surveys [12-16]. The basic idea of multiple imputation is to replace each non-detect with a vector of more than two plausible values from the predictive data distribution [13-15]. In general, multiple imputation results in unbiased estimates, uses all available data, preserves both sample size and statistical power, and reflects the sampling variability. After imputation, any statistical software designed for analyzing complete data can be used.

In our multiple imputation method, we considered non-detect cotinine measurements as missing values based on an imputation model derived from multivariate normal distribution used to construct the predictive distribution for non-detected serum cotinine that included: homocysteine, serum cotinine, age, gender (female/male), race/ethnicity (Non-Hispanic White/Other), and self-reported SHS exposure (yes/no). The number of imputations was set to 10 (i.e. 10 complete datasets including both the detected and imputed non-detected serum cotinine measurements in addition to other variables of interest such as homocysteine, age, gender, race/ethnicity, and self-reported SHS exposure). Ten completed datasets are usually sufficient for multiple imputation [14].

Two different multiple imputation models were used for creating the imputed serum cotinine with LOD. They were both based on a multivariate normal distribution which takes into account the correlation between the variables included in the imputation model. The first model included only homocysteine and serum cotinine; the second imputation model not only included homocysteine and serum cotinine, but also included age, gender, race/ethnicity, and self-reported SHS exposure to take into account the strength of the relationship among all other variables. The imputation model predicted distribution for non-detected serum cotinine from the subjects' homocysteine, age, gender (female/male), race/ethnicity (Non-Hispanic White/Other), and self-reported SHS exposure (yes/no).

The "reverse" Kaplan-Meier method is a non-parametric method that does not require a probabilistic distribution to estimate the survival function from time-to-event type of biological data [17]. It is frequently used with right-censored survival data. For left-censored data, an equivalent estimator can be obtained by Turnbull estimator, which is equivalent to the "reverse" Kaplan-Meier estimator. We performed the "reverse" Kaplan-Meier method by considering left-censored serum cotinine as a "time-to-event" outcome.

Logistic regression is a modelling technique for dichotomous outcomes [18]. We fitted a logistic regression model using dichotomized serum cotinine (below vs. above LOD) as the outcome variable, and the continuous homocysteine values as a predictor variable.

In all of the methods, the univariate regression models for serum cotinine included only continuous homocysteine values as a predictor. The multiple regression models not only included continuous homocysteine values, but also age, gender (female/male), race/ethnicity (Non-Hispanic White/Other), and self-reported SHS exposure (yes/no).

### Statistical analysis

To illustrate the differences in the estimates obtained among different analytical methods for handling LOD, study variables were regressed on the inflammatory marker homocysteine. The NHANES survey weights were not incorporated in any of these analyses to show the differences between the methods as a case study under a random sampling schema. In this case study, the primary objective was to compare the parameter estimates from univariate and multiple regression models that quantify the relationship between inflammatory marker homocysteine and serum cotinine subject to LOD. The univariate regression models for inflammatory marker homocysteine included only serum cotinine as a predictor variable; the multiple regression models included additional variables (as described above). The regression coefficient estimate of serum cotinine, its standard error, and the ratio of the regression coefficient to its standard error as well as the statistical significance were reported and compared among all the methods. All of the analyses were implemented using SAS version 9.2 for Windows statistical software (SAS Institute Inc., Cary, NC, USA). Statistical significance was attained with p-value < 0.05.

## Results

Table 1 summarizes and compares the characteristics of the different methods considered for analyses of left-censored serum cotinine data. Except for the complete case analysis method, all the methods preserved the sample size. The methods differed on how censored values were treated in the analyses. The complete case analysis method was not suitable since it simply excluded all of the censored cotinine values from the analysis. Logistic regression models were also not suitable methods since it collapsed cotinine values into a dichotomous categorical variable (detectable vs. undetectable).

Methods that replaced censored measurements with imputed values were the single and multiple imputation methods. This happens for both single and multiple imputation when either: 1) a non-detect is substituted with an imputed value; or 2) when more than one LOD exists and all values below the highest LOD are treated as non-detects. The methods inherently able to deal with multiple LOD were the multiple imputation, "reverse" Kaplan-Meier method, and logistic regression. Based on the three criteria listed in table 1, the "reverse" Kaplan-Meier method was the most efficient method because it did not require transformation of data to a categorical variable, did not replace censored data with an imputed value, and was able to handle data with multiple LODs.

Parameter estimates and statistical significance for serum cotinine varied according to different methods (Table 2). All the methods found positive associations between serum cotinine and homocysteine in both univariate and multiple regression models. In all methods of analyses, univariate regression estimates (range: 0.001 -- 0.524) were smaller than multiple regression estimates (range: 0.020 -- 1.093). The multiple imputation method reported the smallest estimates in both the univariate and multiple regression models, while the logistic regression models resulted in the largest regression estimates. The only method that did not reveal statistically significant associations between serum cotinine measurements and levels of homocysteine was multiple imputation.

**Table 2:** Association between Homocysteine and Serum Cotinine in Non-Smokers: Results by Different Analytical Methods for Analyzing Left-Censored Biomarker Data

Total n = 9,488		Regression Models					
		
	#subjects included	Univariate*			Multiple*		
		
Method		Estimate (SE)	Estimate/SE	p-value	Estimate (SE)	Estimate/SE	p-value
Complete Case	5,865	0.027 (0.011)	2.455	0.0137	0.053 (0.009)	5.889	< 0.0001

Single Imputation with							

0	9,488	0.055 (0.011)	5.000	< 0.0001	0.083 (0.008)	10.375	< 0.0001

LOD	9,488	0.053 (0.011)	4.818	< 0.0001	0.079 (0.009)	8.778	< 0.0001

LOD/sqrt(2)	9,488	0.054 (0.011)	4.909	< 0.0001	0.081 (0.009)	9.000	< 0.0001

Multiple Imputation	9,488	0.001 (0.025)	0.040	0.9787	0.020 (0.026)	0.769	0.4367

Logistic Regression***	9,488	0.524 (0.055)	9.527	< 0.0001	1.093 (0.076)	14.382	< 0.0001

"Reverse" Kaplan-Meier ***	9,488	0.012 (0.015)	0.800	< 0.0001	0.222 (0.017)	13.059	< 0.0001

LOD: limit of detection; sqrt: squared root

Estimate (SE): regression coefficient and its standard error between Serum Cotinine and Homocysteine

*Univariate regression models for inflammatory marker Homocysteine include serum cotinine only as a covariate; Type-I error rate = 5%.

**Multiple regression models for inflammatory marker Homocysteine include serum cotinine, age in years, gender (female/male), race/ethnicity (non-Hispanic White/Others), and second hand smoking status (yes/no) as covariates; Type-I error rate = 5%.

*** Outcome of these methods is left-censored serum cotinine.

## Discussion

The results of this analysis suggest that different statistical methods for handling LODs can result in variable parameter estimates with resulting p-values both above and below the level of 5% when investigating the associations between serum cotinine and the inflammatory marker homocysteine. While all methods revealed positive associations between serum cotinine and homocysteine, the multiple imputation method did not yield significant parameter estimates. The complete case analysis, single imputation, "reverse" Kaplan-Meier, and logistic regression demonstrated statistically significant positive associations between cotinine and homocysteine levels.

Significant variation between methods was seen in the size of the estimates using both univariate and multiple regression models; a 524 and 55 fold difference was seen in the size of estimates between the multiple imputation and logistic regression using univariate and multiple regression models, respectively. The smallest ratio (estimate/SE) was observed with multiple imputation, while the largest in the logistic regression model. Single imputation using different values for substitution also resulted in slight variation in the size of both univariate and multiple regression models; regression estimates increased from 0.053 to 0.055 and from 0.079 to 0.083 for univariate and multiple regression models, respectively, as the imputation value was changed from the LOD to 0.

Since the true regression coefficient parameter is not known, we do not know for sure which result is the most accurate. Multiple imputation, although believed to be appropriate for complex sample survey data and endorsed in the past for use with NHANES data, is subject to many of the same limitations as complete case and single imputation analysis due to the replacement of censored values with values that were not measured directly [19]. While the creation of binary (below vs. above LOD) or ordinal (below LOD, above LOD as low, medium, high) categorical data from censored data does not violate any assumptions of logistic regression, such statistical methods have much less power compared to "reverse" Kaplan-Meier due to aggregating biomarker measurements into subgroups. Additionally, the grouping and subsequent loss of statistical power with the creation of categorical/ordinal cotinine variables may not be able to detect small associations, such as with the health effects of very low levels of SHS exposure. The "reverse" Kaplan-Meier method, however, appears to be both the most efficient and the most accurate method for this analysis since it is inherently able to handle censored data with multiple LODs, and it avoids the need for data manipulation (needed with imputation methods or logistic regression).

## Conclusions

Researchers who use tobacco smoke exposure biomarkers, such as serum cotinine, should be aware that commonly used analytic techniques for handling left-censored data may bias study results by using suboptimal statistical methods, especially in studies investigating the health effects of SHS exposure due to typically small effect sizes. Analysis of left-censored data requires special attention, as different methods may yield different results as shown in our case study. Many commonly used statistical methods do not properly handle left-censored serum cotinine data because these methods either exclude subjects with undetectable cotinine levels or impute values that are treated as actual observed values. Our analysis suggests "reverse" Kaplan-Meier is the preferred method of analysis of serum cotinine with censored data and with multiple LODs until more in depth research on optimal methods of analysis for left-censored data finds its place in the scientific literature.

Statistical simulation studies are needed to compare the results and to generalize the conclusions to other surveys, as well as to other left-censored biomarker data. In statistical simulation studies, one can compare the estimates obtained from different methods with the true estimates to understand bias and error introduced by varying the different scenarios (such as the percentage of LOD, the magnitude of the correlation between the left-censored biomarker, and the other variables of interest).

Considering the frequent use of SHS biomarkers in public health research and their impact on public health policy, research is needed into the most appropriate statistical methods for use with SHS biomarkers, and well-defined guidelines need to be developed for analyzing SHS biomarkers data.

## Competing interests

The authors declare that they have no competing interests.

## Authors' contributions

TKS and JDC contributed equally to the manuscript. TKS managed the data, performed all statistical analysis and wrote the manuscript. JDC managed the data and wrote the manuscript. LEF and DJL assisted in the writing of the manuscript. All authors read and approved the final manuscript version.
